# A new partial loss of function allele of *rad-54.L*

**DOI:** 10.17912/micropub.biology.000637

**Published:** 2022-09-29

**Authors:** Chantal C Akerib, Rayka Yokoo, Emmanuel T Nsamba, Liesl G Strand, Kei Yamaya, Anne M Villeneuve

**Affiliations:** 1 Departments of Developmental Biology and Genetics, Stanford University School of Medicine, Stanford, CA 94305, U.S.A.

## Abstract

RAD-54.L is required for the repair of meiotic double-strand DNA breaks (DSBs), playing an essential role in promoting removal of recombinase RAD-51 and normal completion of meiotic recombination. Failure to complete meiotic DSB repair leads to 100% lethality of embryos produced by *rad-54.L* null mutant mothers. Here we report a new partial loss of function allele, *rad-54.L(me139)* , that may prove useful for investigating meiotic mechanisms by providing a sensitized genetic background that reduces but does not eliminate the essential functions of RAD-54.L.

**Figure 1. Nature of the rad-54.L(me139) mutation:**
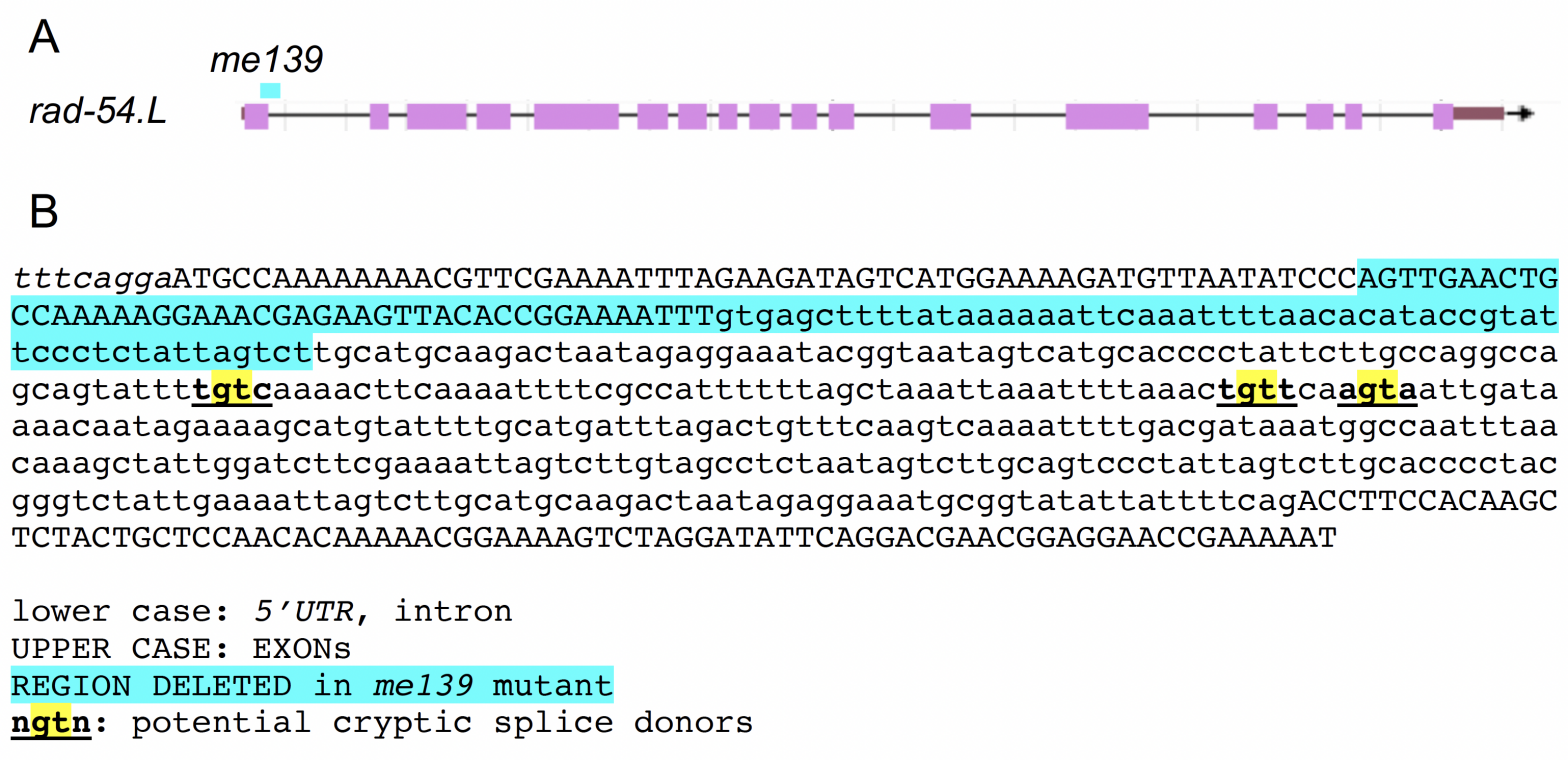
**A.** Schematic of the *rad-54.L* gene structure taken as a screenshot from WormBase WS285, indicating the position of the 102 nt deletion in the *me139* mutant. **B.** Sequence context of the *me139* deletion. UTR and intron sequences are indicated in lower case, exon sequences in upper case. The nucleotides deleted in the *me139* mutant are indicated by cyan highlighting, and potential cryptic splice donor sequences that could be used to generate modified proteins retaining key conserved protein domains are indicated as depicted on figure.

## Description

The *rad-54.L(me139)* mutation was isolated in a modified version of the previously-described “Green Eggs & Him” EMS screen for meiotic mutants based on mis-segregation of *X* chromosomes (Kelly *et al.* 2000). The causative mutation was mapped to chromosome *I* by outcrossing with worms carrying the *tmC18* balancer chromosome (marked with the red pharynx marker *tmIs1236* (Dejima *et al.* 2018)). Homozygous *me139* mutant worms exhibit a variable number of DAPI-stained bodies in diakinesis oocytes and produce many inviable embryos (“maternal-effect lethal” or Mel phenotype), but they also produce some viable progeny that survive to adulthood (15.2% survivors, n = 961), many of which are males (16% males, n = 146; the “high incidence of males” or Him phenotype). Three-factor mapping of the Him/Mel phenotype using *dpy-5* and *unc-29* markers indicated a map position near or to the right of *unc-29* (11/11 Dpy non-Unc recombinants picked up the Him/Mel mutation; 7/7 Unc non-Dpy recombinants did not). Consistent with this mapping, whole genome sequencing of DNA from pooled homozygous mutant recombinants revealed a candidate deletion in the *rad-54.L* gene (formerly called *rad-54* (Mets and Meyer 2009)). This deletion was confirmed by PCR and Sanger sequencing, which identified the lesion as a 102 nt deletion that removes nucleotide residues I..9065146 - I..9065247, thereby truncating the first coding exon and eliminating the adjacent splice donor sequence and a portion of the adjacent intron. Finally, *rad-54.L(me139)* was confirmed to be the causative mutation based on failure to complement the Mel phenotype of *rad-54.L(me98)* , a *rad-54.L* null allele (Roelens *et al.* 2019), as no viable progeny resulted from the embryos produced by *me139 / me98* heterozygous hermaphrodites. As worms homozygous for the *rad-54L* null allele produce 100% dead embryos (Roelens *et al.* 2019), we infer that *rad-54.L(me139)* does not eliminate RAD-54.L function but instead results in a partial loss of function. Perusal of the mutant sequence indicates a capacity to produce several potential modified versions of the RAD-54.L protein that could retain partial function. For example, there are several potential cryptic splice donor sequences that could be used to generate in-frame translatable mRNAs encoding proteins missing only amino acids 21-35 of the normal RAD-54.L protein (with 43, 60, or 62 extra amino acids inserted). As the affected portion of the protein is N-terminal to the highly conserved ATP binding and enzymatic domains that define the protein superfamily, it is plausible that such altered proteins could be at least partially functional. This partial loss of function allele could prove useful for investigating mechanisms of meiotic chromosome inheritance.

## Reagents

**Table:** 

**Strain**	**Genotype**	**Available from**
AV1138	*rad-54.L(me139) / tmC18[dpy-5(tmIs1236)] I* NOTE: Two transgene insertions from the original screening strain are floating in the background of this strain: *ieSi11[EmGFP::syp-3] II* and *ieSi21[sun-1::mRuby] IV*	AV lab
AV1238	*rad-54.L(me139) / tmC18[dpy-5(tmIs1236)] I; ieSi21[sun-1::mRuby] IV* NOTE: *rad-54.L(me139)* homozygotes derived from this strain were used to quantify progeny viability and male frequency	AV lab

## References

[R1] Dejima K, Hori S, Iwata S, Suehiro Y, Yoshina S, Motohashi T, Mitani S (2018). An Aneuploidy-Free and Structurally Defined Balancer Chromosome Toolkit for Caenorhabditis elegans.. Cell Rep.

[R2] Kelly KO, Dernburg AF, Stanfield GM, Villeneuve AM (2000). Caenorhabditis elegans msh-5 is required for both normal and radiation-induced meiotic crossing over but not for completion of meiosis.. Genetics.

[R3] Mets DG, Meyer BJ (2009). Condensins regulate meiotic DNA break distribution, thus crossover frequency, by controlling chromosome structure.. Cell.

[R4] Roelens B, Zawadzki K, Villeneuve A. 2019. me98 is a new allele of rad-54. MicroPubl Biol 2019:10.17912/micropub.biology.000108. 10.17912/micropub.biology.000108 PMC7252391 32550460

